# Acoustic Power Measurement and Thermal Bioeffect Evaluation of Therapeutic Langevin Transducers

**DOI:** 10.3390/s22020624

**Published:** 2022-01-14

**Authors:** Jinhyuk Kim, Jungwoo Lee

**Affiliations:** Department of Electronic Engineering, Kwangwoon University, Seoul 01897, Korea; busl.jhkim@gmail.com

**Keywords:** Langevin transducer, bioeffect, thermal index, acoustic power, thermal ablation

## Abstract

We recently proposed an analytical design method of Langevin transducers for therapeutic ultrasound treatment by conducting parametric study to estimate the effect of compression force on resonance characteristics. In this study, experimental investigations were further performed under various electrical conditions to observe the acoustic power of the fully equipped transducer and to assess its heat-related bioeffect. Thermal index (TI) tests were carried out to examine temperature rise and thermal damage induced by the acoustic energy in fatty porcine tissue. Acoustic power emission, TI values, temperature characteristics, and depth/size of thermal ablation were measured as a function of transducer’s driving voltage. By exciting the transducer with 300 V_pp_ sinusoidal continuous waveform, for instance, the average power was 23.1 W and its corresponding TI was 4.1, less than the 6 specified by the Food and Drug Administration (FDA) guideline. The maximum temperature and the depth of the affected site were 74.5 °C and 19 mm, respectively. It is shown that thermal ablation is likely to be more affected by steep heat surge for a short duration rather than by slow temperature rise over time. Hence, the results demonstrate the capability of our ultrasonic transducer intended for therapeutic procedures by safely interrogating soft tissue and yet delivering enough energy to thermally stimulate the tissue in depth.

## 1. Introduction

Langevin-type transducers, invented by Paul Langevin in 1922, have been widely used to amplify ultrasonic energy in various fields, including the food industry [1,2], sonochemistry [3], and medical practices [4,5]. Typical Langevin transducers are composed of central bolts, rear/front masses, piezoelectric stacks, acoustic boosters, and attachable tools. Ring-shaped piezoelectric elements are clamped between the metal masses by a compression bolt, and an auxiliary acoustic rod may be coupled to the front mass. A few analytical studies have been undertaken to derive systematic design principles of such power transducers in many applications [6,7]. In our previous work, a theoretical design method was proposed via equivalent circuit models to estimate the transducer’s resonance characteristics for therapeutic ultrasound [8]. Mason’s model was employed to represent complex electro-mechanical properties of the piezoelectric ceramics, while T-shaped lumped circuit model was used to describe other passive metal parts by varying intrinsic properties of each material such as acoustic impedance and structural feature. In order to evaluate the effect of bolt tightening on the resonance pattern, more recently, we included a compressive force term applied with a metal bolt as a physical factor in the design platform [9]. Consequently, our numerical results compared with experimental data demonstrated that the proposed model might be used to quantify the resonance signature of a given transducer specification within moderate tolerance.

Besides our previous studies, however, little experimental investigation has been performed to determine the transducer’s capability associated with biological effect under various electrical driving conditions. In this paper, a Langevin transducer was thus fabricated according to our analytical model and excited at actual resonance frequencies. After comparing the resonance property of the transducer with simulation results, we measured the output power emitted from the transducer via an ultrasonic power meter as a function of excitation voltage. Thermal index (TI) is presented to quantify possible thermal risk on fatty porcine tissue induced by acoustic energy. In addition, heat damage caused by acoustic energy on the tissue was evaluated by probing the depth and size of affected sites. Subsequently, in the following sections, further test results are presented to show temperature rise generated by intense ultrasonic energy in similar therapeutic environment.

## 2. Materials and Methods

### 2.1. Design and Fabrication of Langevin Transducer

**Figure 1:**
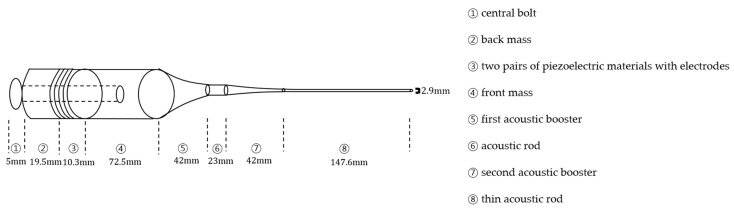
Illustration of Langevin transducer.

Figure 1 shows that rear mass, piezoelectric layers, front mass, acoustic boosters, or horns with attachable rods are clamped by a pre-stress central bolt represented by dotted lines to exert compressive force on each component.

Stainless steel (SUS 304) with high mechanical durability and fatigue strength is selected as the bolt because it can readily fix acoustic parts and prevent their destruction. Given that the back mass facilitates the forward wave propagation by reflecting backward energy, SUS 304 is also preferred due to the fact that it has higher acoustic impedance, i.e., 47 MRayls, than that of piezoelectric elements. It is well known that an even number of piezoelectric elements is required in the case of Langevin transducers [10]. In our model, four pieces of PZT-4 are used to generate longitudinal vibration. In order for the front mass to efficiently transmit acoustic energy towards the forward direction, its acoustic impedance should be lower than that of PZT-4. Aluminum was thus chosen as the front mass since it is durable and has cost-effective properties along with ease of manufacturing. Acoustic boosters with smooth decaying profile are shaped with an exponential outline, making them straightforward to numerically model and fabricate with durability. Thin acoustic rods are intended to interrogate small areas of the targeted tissue with focused ultrasonic power. The overall length of the transducer follows the half-wavelength principle, a systematic design rule to determine the longitudinal dimension of transducer parts [11]. To this end, it is assumed that the resonance frequency of the transducer is 38 kHz. Furthermore, the MATLAB simulation is performed to estimate the electrical input impedance spectrum near the resonance frequency range.

**Figure 2:**
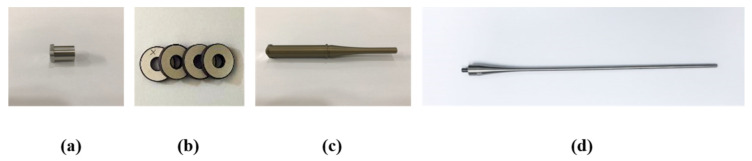
Transducer components: (**a**) back mass; (**b**) piezoelectric elements; (**c**) front mass; (**d**) acoustic rod.

Back mass, PZT-4 stack, front mass with booster, and metal rod are processed by lathe work, as shown in Figure 2.

**Figure 3:**
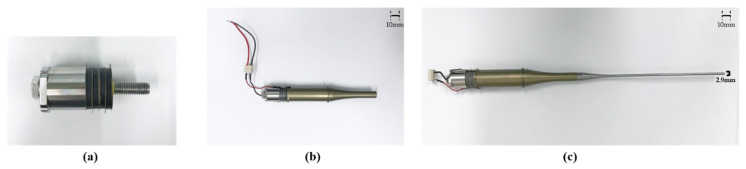
Transducer assembly: (**a**) back mass and piezoelectric layer clamped by a central bolt; (**b**) transducer without acoustic rod; (**c**) complete transducer set.

Anodizing treatment for the front mass is further performed to improve corrosion resistance under liquid environment. To amplify the acoustic power along the longitudinal direction, the decaying rate of each cross-sectional area in both boosters was designed to be 16.5 m^−1^ and 29.6 m^−1^, respectively. Four ring-type PZT-4 (C-203, Fuji Ceramic Corporation, Fujinomiya, Japan) pieces were sandwiched between two solid masses, where five 0.5 mm copper-nickel layers were also electroplated to feed sinusoidal input to PZT-4. The aforementioned components are combined through stainless steel bolts with 12 N·m torque loaded by a digital torque-wrench (WPC3-030, Bluetec, Taichung, Taiwan). Acoustic rods are made of titanium that has widely been used for therapeutic purposes due to its good biocompatibility with the human body. The assembly procedure of the transducer set is then represented in Figure 3.

### 2.2. Calibration of Acoustic Pressure

**Figure 4:**
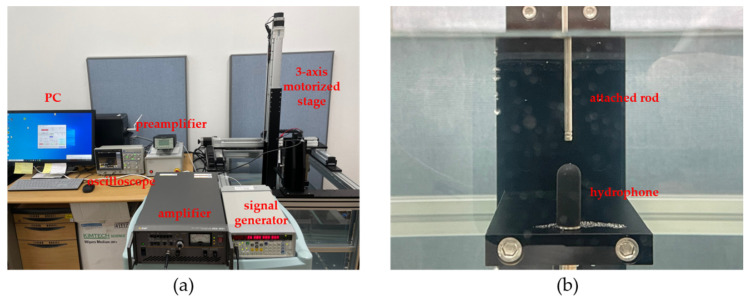
Pressure calibration system: (**a**) overall set-up; (**b**) hydrophone, and titanium rod.

The hydrophone system for characterization of the acoustic field was composed of a water bath, 3-axis motorized stage, manual stage, hydrophone (8103, Bruel & Kjaer, Naerum, Denmark), preamplifier (2692-0S1, Bruel & Kjaer, Naerum, Denmark), signal generator (SG382, Stanford Research System, Sunnyvale, CA, USA), RF amplifier (HAS 4051, NF, Yokohama, Japan), oscilloscope (DS-5652, IWATSU, Kugayama, Japan), and PC. A sinusoidal signal with amplitude ranging from 1 V peak-to-peak (V_pp_) to 1.5 V_pp_ at 38 kHz was produced from a signal generator and augmented by an RF amplifier with variable gain from 20 to 200. The tip of the excited transducer was immersed in the water bath by controlling a manual stage equipped with a digital length meter. The hydrophone sensor was translated with a motorized positioning stage for measuring spatial acoustic field. The pre-amplified received RF signal was transmitted to PC for calibration. The whole experimental set-up and enlarged view of the hydrophone with titanium rod are presented in Figure 4. Because the tip diameter of our transducer was 2.9 mm, much smaller than the hydrophone surface coated with acoustic absorber of convex shape, most of the incident wavefronts on the surface are likely to be deflected away, if any, and few are reflected back to the transducer. Despite the fact that standing wave formation could still be possible for Langevin transducers [12], we assume that their effect on vibration characteristics would be minimal here.

### 2.3. Acoustic Power Measurement

**Figure 5:**
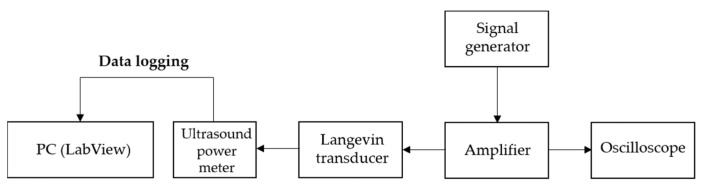
Layout of acoustic power measurement system.

**Figure 6:**
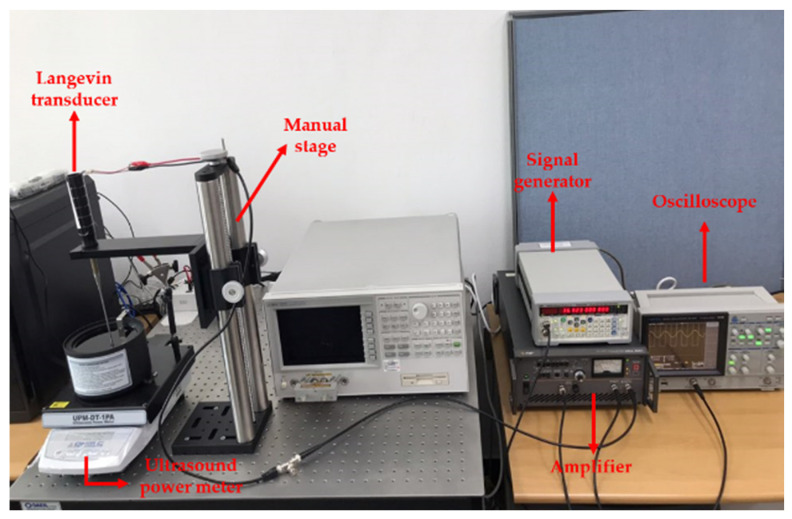
View of power measurement system.

A system configuration of power measurement is illustrated in Figure 5. Thirty-eight kilohertz continuous sinusoidal signals from the signal generator were augmented by the amplifier with the same gain as in pressure calibration. Such amplified signals were subsequently input to the transducer clamped by a manual stage and sent to the oscilloscope for simultaneously monitoring the measurement progress. Ultrasound power meter (UPM-DT-1PA, Ohmic Instrument, St. Charles, MO, USA) was employed to calibrate the energy detected from an absorber target in water bath. Finally, LabView control panel was used to record the emitted power in real-time. The experimental set-up is shown in Figure 6.

### 2.4. Tracking of Temperature Variation and Evaluation of Thermal Index (TI)

**Figure 7:**
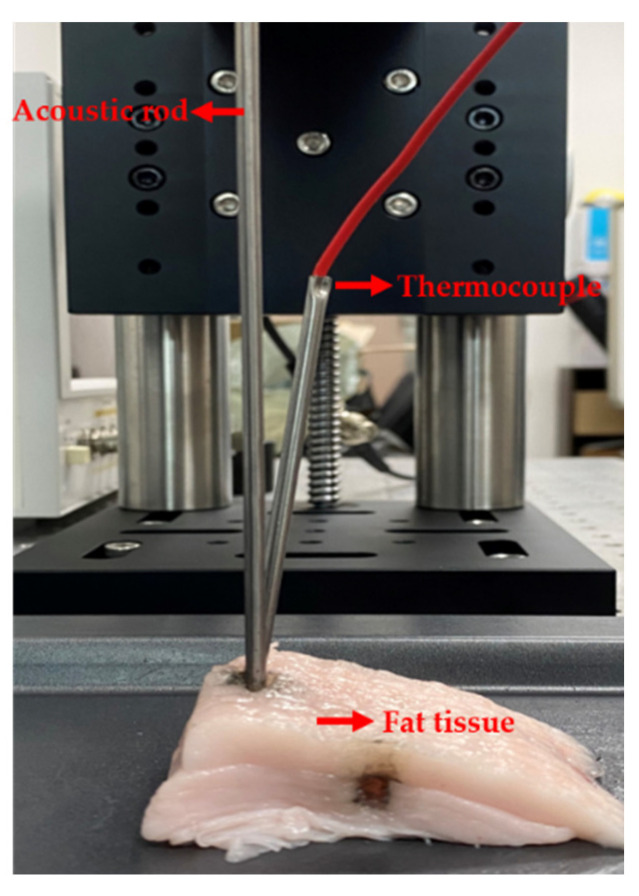
Experimental view for temperature tracking and TI.

A schematic diagram for temperature change in porcine tissue is given in Figure 7. Both thermocouple (J-type) and data logger (EL-USB-TC, LASCAR electronics, Erie, PA, USA) were installed to record temperature distribution in the tissue. The acoustic rod punctured the tissue to 10 mm, deep enough to fully transmit the radiation energy to the tissue. As the peak-to-peak magnitude of excitation voltage varies from 100 V_pp_ to 300 V_pp_, the transducer examines the tissue in several distinct locations at the same depth.

### 2.5. Thermal Index (TI)

Typical ultrasound equipment provides users and patients with a thermal index (TI) value during ultrasonic exposure. TI value makes the operator estimate the thermal effect on the human body, especially temperature rise in soft tissue. TI is determined by the ratio of the average acoustic power to the attenuated acoustic power required in increasing temperature at a particular point by 1 °C, as represented in Equation (1) for scan mode [13] and for non-scan mode in Equation (2) [14], where W and $f_{res}$ are acoustic output power and resonance frequency of a given transducer. The Food and Drug Administration (FDA) regulates that the maximum *TI* is 6 in order to prevent excessive temperature rise from damaging tissues [15]. Since the various parameters, such as attenuation and the absorption coefficient, are not included in the *TI* estimate, a particular index only refers to potential tissue heating, not an exact temperature rise [14,16].

(1) $$TI=\frac{W_{0}}{W_{\deg}}$$

(2) $$TIS=\frac{W\cdot f_{res}[\mathrm{MHz}]}{210[\mathrm{mW}]}$$

## 3. Results and Discussion

**Figure 8:**
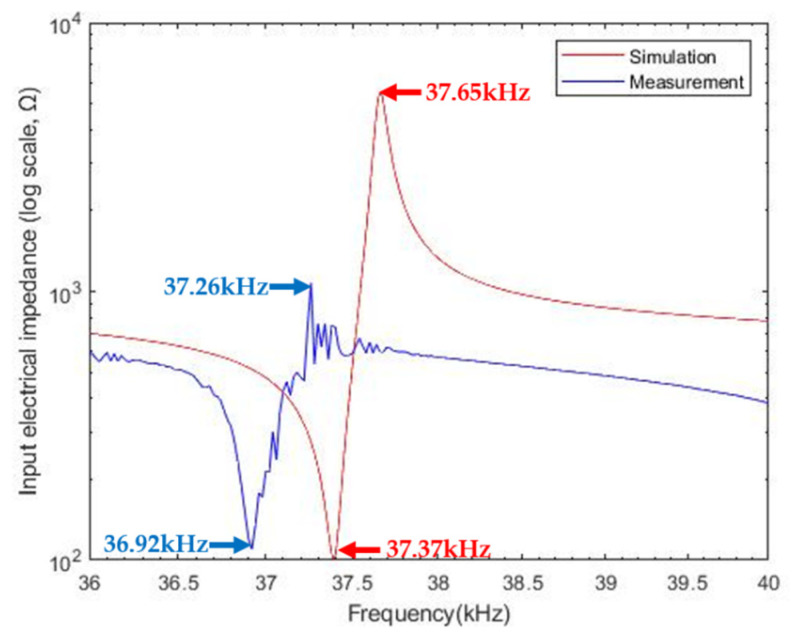
Magnitude of input impedance: simulation and measurement.

Here, the frequency response of input electrical impedance for the transducer was measured with an impedance analyzer (4294A, Agilent, Santa Clara, CA, USA) and compared with our design estimate by MATLAB simulation. When the surgical rod was attached to the transducer, resonance characteristics of the impedance were shown to be between 36 kHz and 40 kHz in Figure 8. In simulation, resonance (*f_r_*) and anti-resonance frequencies (*f_a_*) were identified at 37.4 kHz and 37.7 kHz, where their corresponding impedances were 101 kΩ and 5.4 kΩ, respectively. In an experiment, *f_r_* and *f_a_* were seen to be 36.9 kHz and 37.3 kHz, and those impedances were 109 Ω and 1.1 kΩ. The anti-resonance peak difference between model and test results was 4.3 kΩ, which may have arisen from undesirable shear vibration of the auxiliary metal rod. 

**Figure 9:**
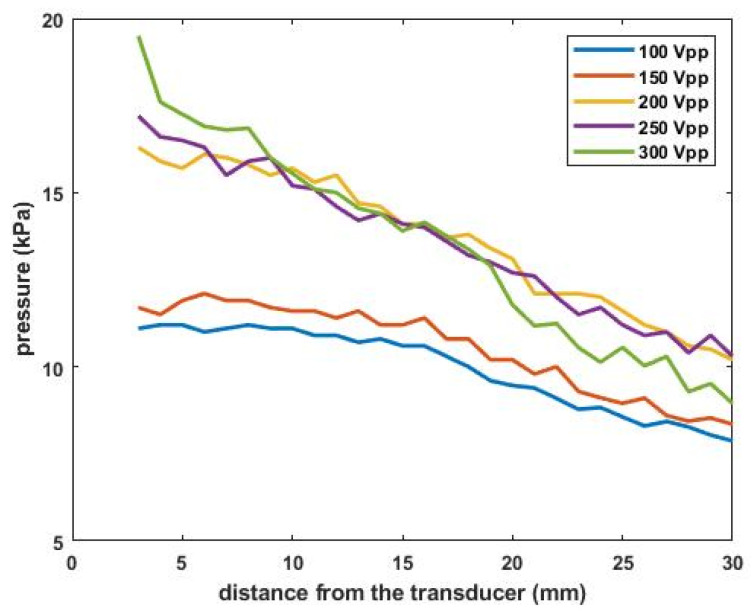
Acoustic pressure measurement results.

Results of pressure calibration are shown in Figure 9. To prevent direct damage to the hydrophone, the measuring distance between the sensor and the transducer tip was set from 3 mm to 30 mm with 0.5 mm increments. The number of measurements in each condition was 5. The applied AC voltage to the transducer was varied from 100 V_pp_ to 300 V_pp_ with 50 V_pp_ increments. Since the measured *f_r_* was 36.9 kHz as mentioned above, the transducer is excited by continuous sinusoidal waves close to that frequency. In general, the magnitude of the pressure was proportional to the driving voltage. The maximum pressure was 19.5 kPa with 300 V_pp_, and the minimum value was 11.1 kPa with 100 V_pp_. These results imply that since the sound propagates in forward direction, more attenuated energy is picked up with the hydrophone as the distance from the transducer increases. In particular, a sharp drop in pressure amplitude was seen at 300 V_pp_, under which nonlinear sound formation becomes dominant. This may be because the cumulative nonlinear effect steepens the waveform and drastically reduces the amplitude of the pressure [17,18].

**Figure 10:**
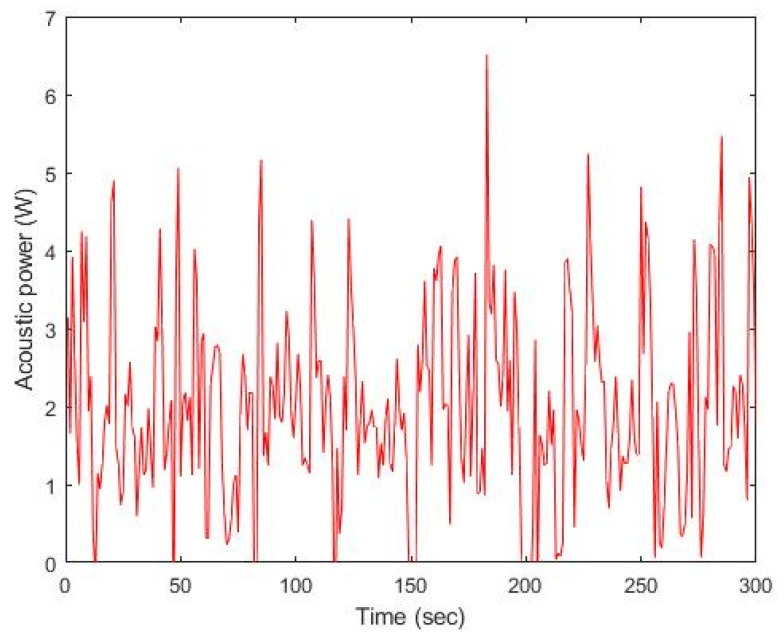
Temporal acoustic power in the case of 100 V_PP_ excitation.

Acoustic power was measured by varying the voltage amplitude for transducer excitation from 100 V_pp_ to 300 V_pp_. Input voltage from the signal generator was adjusted by the amplifier to generate 50 V_pp_ increments, and the transducer was fed by a continuous sinusoidal signal at 37 kHz for 300 s. The maximum power (P_max_), minimum power (P_min_), temporal peak power (P_peak_), and temporal average power (P_avg_) were computed, where P_avg_ was employed to determine TI. The temporal acoustic power for 100 V_pp_ is given in Figure 10. Average values of P_min_, P_peak_, and P_avg_ are 0.03 W, 6.50 W, and 2.00 W, respectively. The resultant TI is 0.4.

**Figure 11:**
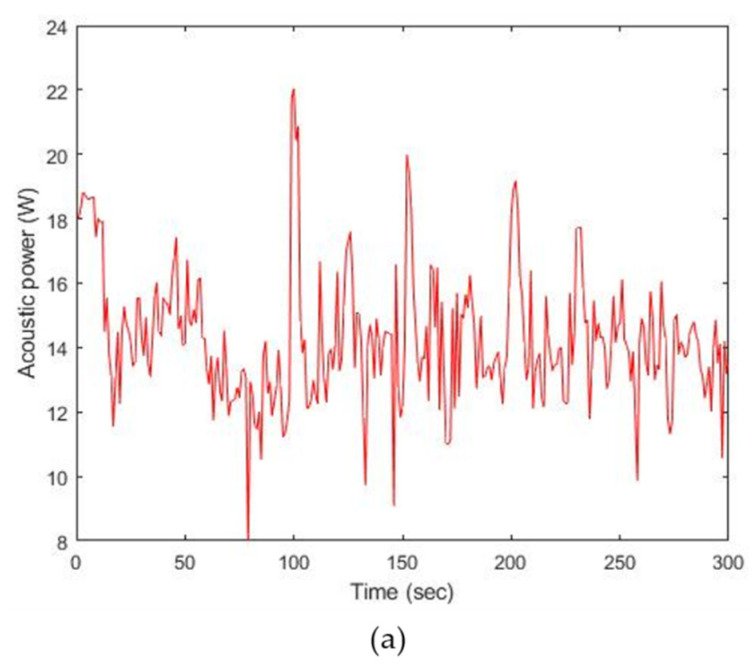

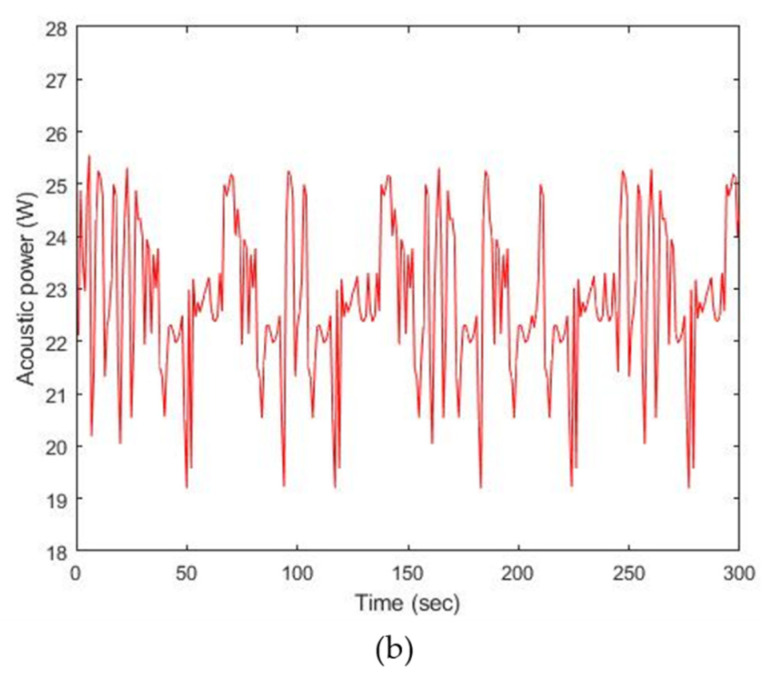
Results of acoustic power emission: (**a**) 200 V_pp_, (**b**) 300 V_pp._

Likewise, Figure 11 shows the test results of output power with 200 V_pp_ and 300 V_pp_. According to Figure 11a, P_min_, P_peak_, and P_avg_ are 7.9 W, 22.0 W, and 14.3 W, and the corresponding TI is 2.5. For 300 V_pp_ excitation, these power values are 19.9 W, 26.5 W, and 23.1 W, as seen in Figure 11b, where TI is 4.0. These results indicate that both output power and TI increase as the applied input amplitude becomes greater. There are fluctuations in the recorded power regardless of the amplitude of the excitation signal. Since consistent vibrational behavior produces the heat generation in the piezoelectric material, which leads to the *f_r_* decrease, the resonance tracking procedure must be needed [19,20]. Due to the procedure, detection of current and computation of phase in the operation system result in inevitable fluctuations in output power.

**Figure 12:**
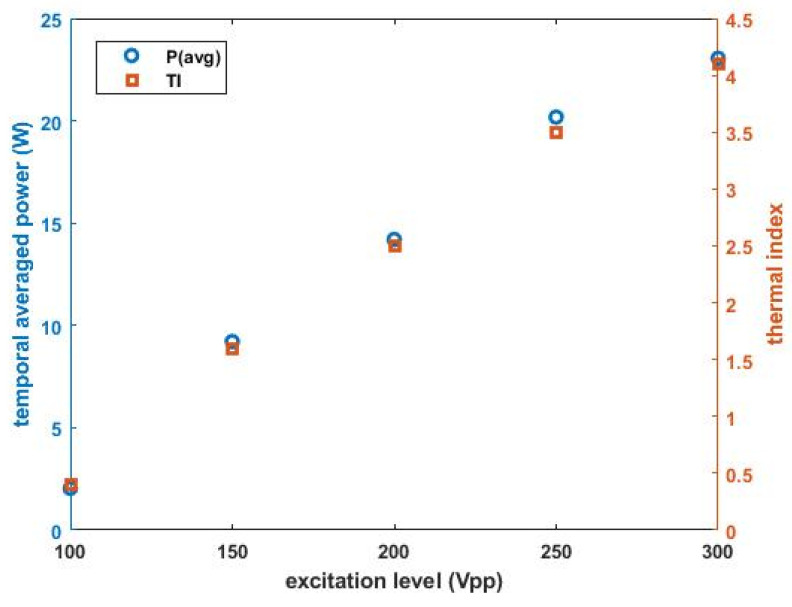
Temporal average power output of the transducer as a function of excitation voltage.

P_avg_ values and their corresponding TIs are represented as a function of voltage amplitude in Figure 12. P_avg_ from the transducer loaded by 150 V_pp_ amplitude is 9.2 W, and the corresponding TI is 1.6. For 250 V_pp_ stimulation, those values are 20.2 W and 3.6. The increment in acoustic power and TI gradually decreases as higher voltage is applied. For example, differences in P_avg_ and TI, 7.2 W and 1.3, are maximized between 100 V_pp_ and 150 V_pp_, while they are minimized to be 2.6 W and 0.5 between 250 V_pp_ and 300 V_pp_. This indicates that the output power becomes saturated because our hard-type transducer generally reveals nonlinear behavior under high excitation conditions [21]. Subsequently, these acoustic power levels render the linear raise in TI. The minimum and maximum TIs are 0.4 and 4.1 at 100 V_pp_ and 300 V_pp_, respectively. It is demonstrated that TIs obtained under given conditions here satisfy the FDA regulation regarding ultrasonic exposure over the range of tens of kHz [22,23]. All measured power and TI data are listed in Table 1.

**Figure 13:**
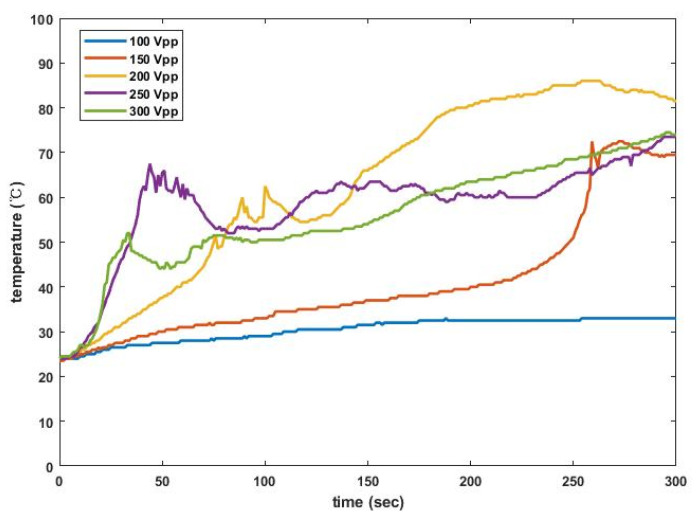
Temperature responses to various electrical driving conditions over time.

In addition, results for temperature dependence on electrical excitation are represented in Figure 13. When the sinusoidal signal with 100 V_pp_ was applied to the transducer, the measured temperature gradually increased from 24 °C to 33 °C for 300 s and left a small area of heat damage. For 150 V_pp_, furthermore, the temperature abruptly surged to 72.5 °C at about 260 s and stayed there. Beyond 200 V_pp_ excitation, the temperature rose fast enough to emulsify the porcine fat after 20 s and reached a saturation level near 60 °C, even 80 °C for 200 V_pp_. Such heat generation describes a common pattern where the temperature after instant rise declines for several seconds and then increases consistently. In fact, this thermal performance may be helpful in removing various tissues including muscles [24] and tumors [25,26]. Except for the100 V_pp_ case, the maximum temperature is often measured to be over 65 °C, high enough to ensure complete destruction of cancer cells [27].

**Figure 14:**
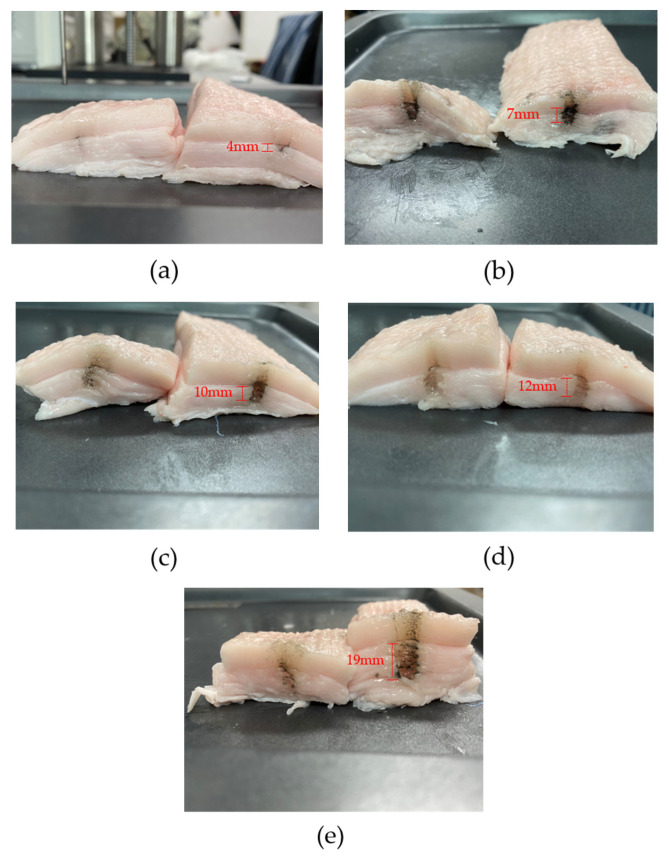
Heat damage afflicted by the transducer as a function of applied voltage: (**a**) 100 V_pp_, (**b**) 150 V_pp_, (**c**) 200 V_pp_, (**d**) 250 V_pp_, (**e**) 300 V_pp._

In order to evaluate the influence of acoustic power emission on our tissue specimen, thermal ablation was visualized by identifying affected region in dark, as illustrated in Figure 14. The depth of the region was approximately 5 mm throughout the test. For 100 V_pp_ application as shown in Figure 14a, the length of the ablated site was 4 mm in axial direction parallel to the sound propagation. In Figure 14b, gradual heat generation for a relatively long time formed a clear dark spot within 7 mm. As depicted in Figure 14c,d, electrical signals with 200 V_pp_ and 250 V_pp_ caused rapid temperature elevation up to 66 °C in a few seconds, charring fatty tissue. The largest damaged site, 19 mm, is observed in Figure 14e when the transducer was driven by the highest voltage in the experiment, namely 300 V_pp_. Due to such an excessive exposure, damaged spots were located more extensively right underneath the tissue surface. According to the above results, therefore, thermal ablation is likely to be more sensitive to steep heat surges rather than to slow temperature rise over time. Hence, it is necessary to consider both temperature rise characteristics and extent of thermal damage at the same time for estimating thermal effect based on acquired TIs. The maximum temperature, T_max_, and the depth of the thermally afflicted area are summarized in Table 2 under each condition.

Since the energy emitted from Langevin transducers is strong enough to influence our sample tissue as reported above, this approach can further be adapted to other applications. In order to prevent undifferentiated stem cells from tumor formation, for instance, localized cell removal can be achieved by Langevin transducers without direct contact with the cell surface [28]. A three-dimensional cell patterning of collateral cylindroids [29] and a scaffold cell seeding technique [30] can similarly be implemented with acoustophoretic force exerted by bolt-clamped transducers. Hence, these dynamic techniques may have advantages over conventional static processes, e.g., perfusion of cell suspension, in a way that specific cell functions can be customized without directly affecting other neighboring cells.

## 4. Conclusions

This work carried out experimental investigations under various excitation conditions to measure the acoustic power of a fully equipped Langevin transducer and to assess its heat-related bioeffect with porcine tissue. To this end, we first built a Langevin transducer according to our design model and excited the sample at actual resonance frequency determined by impedance analyzer. After comparing the resonance property of the transducer with simulation results, we measured the output power from the transducer via an ultrasonic power meter as a function of excitation voltage. The temporal minimum, maximum, and averaged powers were presented to determine TIs along with temperature rise characteristics in the tissue. It is demonstrated that TIs acquired in this study meet the FDA regulations for typical therapeutic ultrasound. Furthermore, heat damage caused by acoustic energy on the exposed tissue was evaluated by probing the depth and size of affected zones. It was found that fast heat elevation promotes tissue removal more effectively and that stronger acoustic power gives rise to greater extent of thermal ablation. It is also shown that such heat-related aspects of Langevin transducers may be quantitatively customized by adjusting physical parameters directly affecting the transducer’s performance. Therefore, the results from this paper justify the use of our Langevin transducer in heat treatment of various biological targets such as muscles and cancer cells.

## Figures and Tables

**Table 1:** Results of acoustic power and TI under different driving conditions.

	100 V_pp_	150 V_pp_	200 V_pp_	250 V_pp_	300 V_pp_
P_min_	0.03	5.46	7.86	18.57	19.90
P_max_	6.50	11.44	22.04	21.5	26.45
P_avg_	2.04	9.21	14.27	20.18	23.05
TI	0.4	1.6	2.5	3.5	4.1

**Table 2:** Maximum temperature and depth of exposed zone.

	100 V_pp_	150 V_pp_	200 V_pp_	250 V_pp_	300 V_pp_
Maximum temperature (°C)	33	72.5	83.5	73.5	74.5
Depth (mm)	4	7	10	12	19
